# Interplay of MKP-1 and Nrf2 drives tumor growth and drug resistance in non-small cell lung cancer

**DOI:** 10.18632/aging.102531

**Published:** 2019-12-06

**Authors:** Hongyan Wang, Kaihua Liu, Zhexu Chi, Xihang Zhou, Guoping Ren, Ren Zhou, Yinyan Li, Xiuwen Tang, Xiu Jun Wang

**Affiliations:** 1 Department of Pharmacology and Cancer Institute of the Second Affiliated Hospital, Zhejiang University School of Medicine, Zhejiang University, Hangzhou 310058, PR China; 2 Department of Biochemistry, Zhejiang University School of Medicine, Zhejiang University, Hangzhou 310058, PR China; 3 Department of Pathology of the First Affiliated Hospital, Zhejiang University School of Medicine, Zhejiang University, Hangzhou 310003, PR China; 4 Institute of Pathology and Forensic Medicine, Zhejiang University School of Medicine, Zhejiang University, Hangzhou 310058, PR China; 5 Department of Pathology and Path-physiology, Zhejiang University School of Medicine, Zhejiang University, Hangzhou 310058, PR China

**Keywords:** mitogen-activated protein kinase phosphatase 1, Nrf2, lung cancer

## Abstract

Alterations in KEAP1/ NF-E2 p45-related factor 2 (NFE2L2/Nrf2) signaling pathway have been reported in 23% lung adenocarcinoma patients, suggesting that deregulation of the pathway is a major cancer driver. Here we report that mitogen-activated protein (MAP) kinase phosphatase 1 (MKP-1) drives tumor growth and drug resistance by up regulating transcription factor Nrf2. In non-small cell lung cancer (NSCLC) cells and xenografts, MKP-1 knockdown triggered the down-regulation of the metabolic enzymes and cytoprotective proteins, which are the target genes of Nrf2. Consequently, the cell growth was markedly inhibited with decrease of tumor metabolisms and GSH contents. Moreover, MKP-1 silencing sensitized NSCLC cells to cisplatin treatment. Mechanistically, MKP-1 inhibited the ubiquitylation of Nrf2 via a direct interaction with the transcription factor. Nrf2 was hence stabilized and its transcriptional program was activated. Notably, Nrf2 elevated MKP-1 expression at transcriptional level. In human lung adenoma tumor samples, high levels of expression of MKP-1, Nrf2, and its target gene heme oxygenase 1 were closely correlated. Thus, MKP-1 and Nrf2 form a forward feedback loop in lung cancer cells, which stabilizing and activating Nrf2 to promote anabolic metabolism and GSH biosynthesis. This study uncovers a novel role of MKP-1 in the malignant evolution of cancers.

## INTRODUCTION

Lung cancer is one of the most common cancers worldwide and ranked the first leading cause of cancer death worldwide [1]. NSCLC comprises about 85% of all lung cancer cases and about half of NSCLC are lung adenocarcinoma (LUAD) [2]. In the past ten years, big progress has been achieved in the treatment of LUAD with targeted lung cancer therapy such as drugs targeting the epidermal growth factor receptor (EGFR) mutation or the anaplastic lymphoma kinase (ALK)-rearranged NSCLC patients group [3]. However, the prognosis of LUAD is still unsatisfactory because of drug resistance, recurrence and metastasis [1]. Therefore, a better understanding of molecular mechanisms underlying tumorgenesis and drug resistance may pave the way for the development of effective therapeutic strategies for LUAD [1–4].

Mitogen-activated protein kinase (MAPK) phosphatase 1 (MKP-1), also known as dual specificity protein phosphatase 1 (DUSP1), is a nuclear mitogen and stress-inducible MKP that is highly expressed in different types of human tumors, including those of the lung, breast, bone, ovary, bladder, prostate, and osteosarcoma [5–8]. MAPKs, the substrates of MKPs, play important roles in proliferation, the stress response, apoptosis, and the immune response. There are three well-known MAPK subfamilies: ERK, c-Jun NH_2_-terminal kinases (JNK), and p38 MAPK isoforms. MAPKs are activated through a cascade of sequential phosphorylation events. The phosphorylation of MAPKs on threonine and tyrosine residues by specific upstream MAPK kinases (MEKs or MKKs) leads to their activation. Conversely, MKPs dephosphorylate MAPKs on tyrosine and threonine residues of the signature T-X-Y motif located within the activation loop of the kinase [9–11]. MKP-1 is able to dephosphorylate ERK, JNK, and p38. There is increasing evidence that MKP-1 may be abnormally up regulated in lung cancer [9–11]. However, whether the overexpression is a cause of, or actually contributes to, the malignant phenotype rather than simply being a consequence of cell transformation is not clear yet.

Nrf2 is a master transcriptional activator of cytoprotective genes; it activates transcription in response to electrophiles and reactive oxygen species [12, 13]. Under normal conditions, Nrf2 is mainly controlled by ubiquitylation mediated by Kelch-like ECH-associated protein 1 (Keap1), and is degraded by the proteasome. Exposure to stimuli inactivates Keap1 and stabilizes Nrf2. Nrf2 translocates into the nucleus, binds to the antioxidant response element (ARE) and activates the transcription of many cytoprotective genes that encode detoxifying enzymes and antioxidant proteins, including aldo-keto reductase B10 (AKR1B10), heme oxygenase 1 (HO-1), NAD(P)H:quinone oxidoreductase 1 (NQO1), aldo-keto reductase C1 (AKR1C1), and glutamate-cysteine ligase, catalytic subunit (GCLC). In contrast to the acute physiological regulation of Nrf2, in neoplasia there is evidence for increased basal activation of Nrf2. Indeed, somatic mutations that disrupt the Nrf2–Keap1 interaction to stabilize Nrf2 and increase the constitutive transcription of Nrf2 target genes have been found in various human cancers [14–18]. For example, somatic gain-of-function mutations of *Nrf2* or somatic loss-of-function mutations of either *Keap1* or *Cullin 3* (*Cul3*) are frequently reported in lung cancer. Recent large scale genomic studies have revealed alterations in components of the Keap1/Nrf2 stress response pathway in 23% of LUADs [19]. Besides regulating cytoprotective genes, Nrf2 induces the expression of metabolic genes, including glucose-6-phosphate dehydrogenase (G6PD), isocitrate dehydrogenase 1 (IDH1), phosphogluconate dehydrogenase (PGD), phosphoribosyl pyrophosphate amidotransferase (PPAT), methylenetetrahydrofolate dehydrogenase 2 (MTHFD2), transketolase (TKT), and malic enzyme 1 (ME1) in non-small cell lung cancer (NSCLC) cells [20, 21]. Cancers with high Nrf2 levels are associated with poor prognosis [14–18, 22, 23], resistance to therapy and rapid proliferation [14–8]. However, the precise mechanism by which Nrf2 promotes tumourigenesis and drug resistance is not clear [19].

Recently, we reported that both the basal and induced Nrf2-antioxidant response was reduced substantially in *Mkp-1^-/-^* mice, and that Mkp-1 protects mice against toxin-induced liver damage by activating Nrf2 cytoprotective system [24]. Our studies showed that direct interaction of the DIDLID motif of NRF2 with Mkp-1 leads to increased Nrf2 stability and positive regulation of the Nrf2 pathway. A role of the Mkp-1/Nrf2 axis in limiting inflammation in murine colitis has also been demonstrated [25, 26]. However, it is not known whether MKP-1 could cross-talk with Nrf2 to support an oncogenic program that increases cell proliferation and drug resistance in NSCLC cells. The present study was therefore carried out to elucidate the potential role of MKP-1/Nrf2 in the context of tumour growth and drug resistance.

## RESULTS

### MKP-1 regulates NSCLC cell growth and tumor metabolism

**Figure 1:**
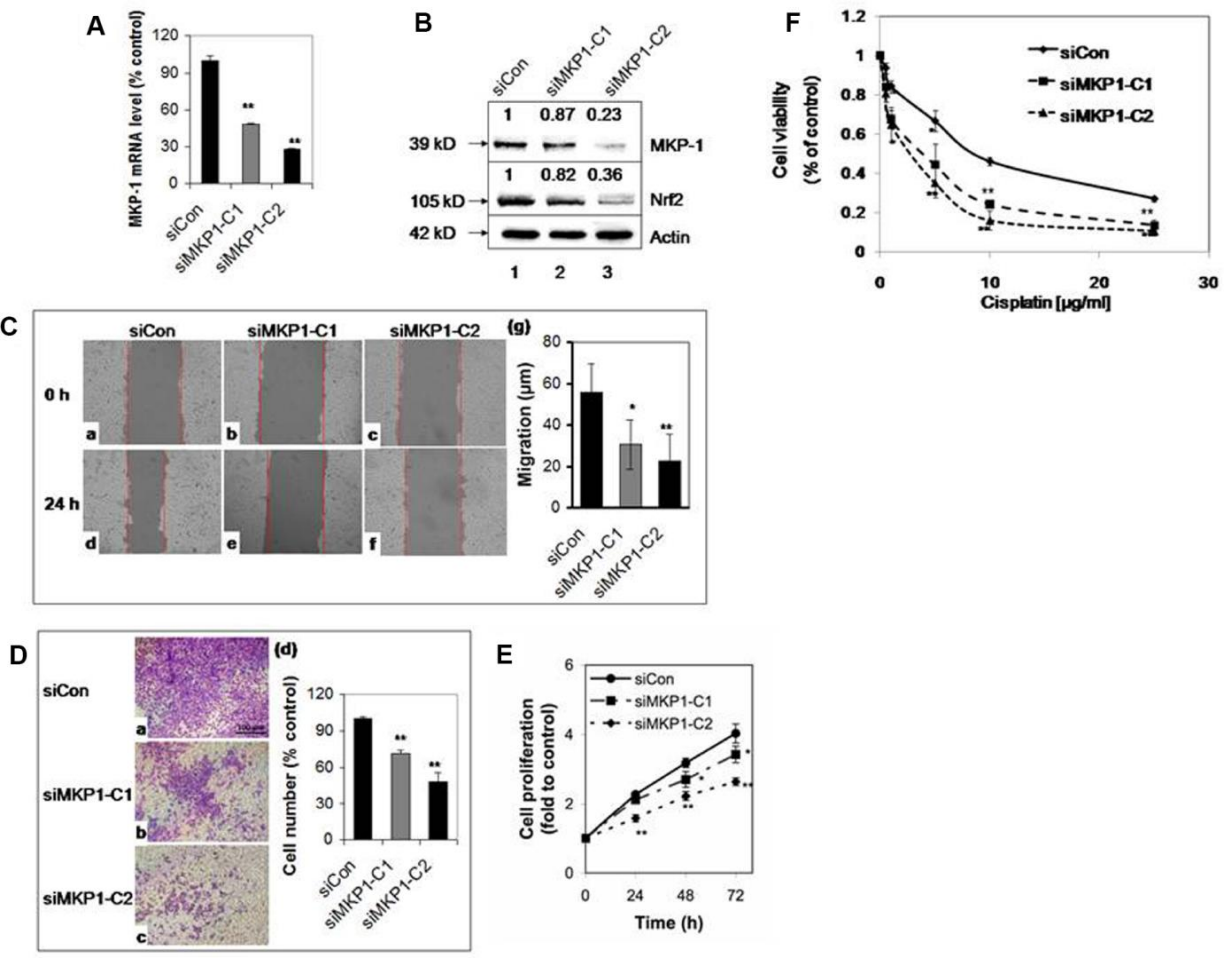
**MKP-1 regulates the proliferation and drug resistance of A549 NSCLC cells.** Two stable MKP-1-knockdown cell lines, siMKP1-C1 and siMKP1-C2, were generated after stable transfection with the pGFP-V-RS-MKP-1 plasmid into A549 cells. siCon expressing empty pGFP-V-RS vector was used as a negative control. (**A**) MKP-1 mRNA levels in siCon, siMKP1-C1, and siMKP1-C2 cells as determined by Taqman RT-PCR. The 18S rRNA level was used as an internal control and the value for siCon cells was set at 100%. Values are mean ± SD, n = 3. (**B**) Immunoblots of whole-cell lysates probed with anti-MKP-1, anti-Nrf2 or anti-actin. The relative levels of MKP-1 and Nrf2 normalized to actin are shown above each lane. Blots are representative three separate experiments. (**C**) Knockdown of MKP-1 reduces cell migration. Scratch assay images of siMKP1-C1 (b and e), siMKP1-C2 (c and f) and siCon cells (a and d) acquired at 0 (a-c) and 24 h (d-f). Red lines define the areas lacking cells. Statistics are shown in (g). Values are mean ± SD, n = 3. (**D**) MKP-1 promotes motility of NSCLC cells. Images of transwell migration assays of siMKP1-C1 (b), siMKP1-C2 (c), and siCon cells (a). (d) Statistics for three experiments. The number of siCon cells was set at 100%. Values are mean ± SD, n = 3. (**E**) Knockdown of MKP-1 decreases cell proliferation. Cells were cultured for 24, 48, or 72 h and the numbers determined by MTS assays. The value for the same cells at 0 h was set at 1. Values are mean ± SD, n = 3. (F) Knockdown of MKP-1 increases sensitivity to cisplatin in NSCLC cells. siMKP1-C1, siMKP1-C2 and siCon cells were exposed to cisplatin (0–25 μg/ml) for 48 h. The cell viability was determined by MTT method. The value of DMSO treatment was set at 1. Values are means ± SD, n = 3. *p <0.05, **p <0.01.

**Figure 2:**
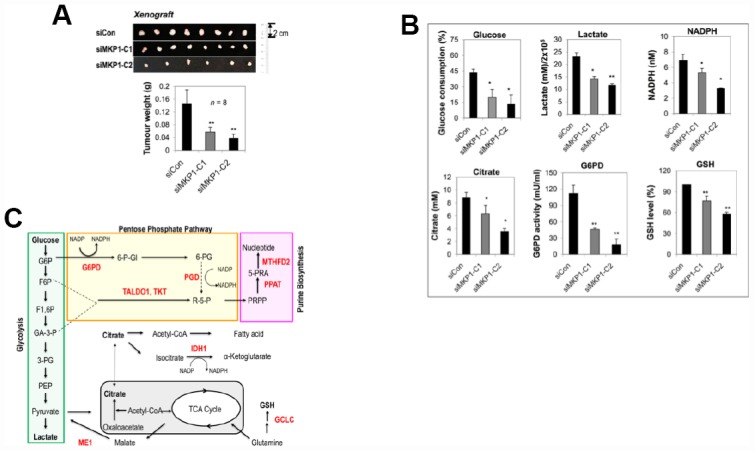
**MKP-1 regulates metabolism of A549 NSCLC cells and promotes tumor growth.** (**A**) Representative images and weights of tumors 6 weeks after subcutaneous injection of siMKP1-C1, siMKP1-C2 or siCon cells into Nu/Nu mice. Values are mean ± SD, n = 8. (**B**) MKP-1 knockdown alters glucose and glutamine metabolism in A549 Cells. siMKP1-C1, siMKP1-C2 and siCon cells were cultured for three days before glucose consumption, lactate production, citrate, NADPH, G6PD activity, and GSH levels were determined. The value for culture medium at day 0 was set at 100% for the analysis of glucose consumption. The value for siCon cells was set at 100% for the analysis of GSH levels. Values are mean ± SD, n = 3, *p <0.05, **p <0.01. (**C**). MKP-1 regulates glucose and glutamine metabolism in NSCLC cells. Metabolite abbreviations: G6P, glucose 6-phosphate; F6P, fructose 6-phosphate; F1,6P, fructose 1,6-bis-phosphate; GA3P, glyceraldehyde 3-phosphate; 3-PG, 3-phosphoglycerate; PEP, phosphoenolpyruvate; 6-P-Gl, 6-phosphogluconolactone; 6-PG, 6-phosphogluconate; R-5-P, ribulose5-phosphate; PRPP, 5-phosphoribosyl-α-1-pyrophosphate; 5-PRA, b-5-phosphorybosylamine; GSH, reduced glutathione. The metabolic enzymes regulated by MKP-1/Nrf2 in glucose and glutamine metabolisms, are shown in red. G6PD, glucose-6-phosphate dehydrogenase; GCLC, glutamate-cysteine ligase, catalytic subunit; IDH1, isocitrate dehydrogenase 1; ME1, malic enzyme 1; MTHFD2, methylenetetrahydrofolate dehydrogenase 2; PGD, 6-phosphogluconate dehydrogenase; PPAT, phosphoribosyl pyrophosphate amidotransferase; TALDO1, transaldolase1; TKT, transketolase. The mitochondrion is shown in gray.

To explore the role of MKP-1 in lung cancer, we developed two MKP-1-knockdown lines from A549 cells, siMKP1-C1 and siMKP1-C2. RT-PCR analysis revealed >50% reduction of MKP-1 mRNA levels in siMKP1-C1 and siMKP1-C2 cells, compared to the control cell line siCon (Figure 1A). Similar reductions in MKP-1 protein expression were also found (Figure 1B). Knockdown of MKP-1 reduced the migration (Figure 1C), invasion (Figure 1D), proliferation (Figure 1E) and drug resistance to cisplatin of siMKP1-C1 and siMKP1-C2 cells (Figure 1F). MKP-1 knockdown also markedly inhibited the tumor growth in siMKP1-C1 and siMKP1-C2 xenografts (Figure 2A). Given that tumor cells often develop an altered metabolism to cope with the demands of increasing cell-mass during growth, we investigated whether the MKP-1-dependent proliferation involves metabolic reprogramming. Indeed, MKP-1 knockdown caused decreased glucose uptake and lactate production, indicating reduced glycolysis (Figure 2B, 2C). Knockdown of MKP-1 decreased the activity of G6PD, the rate-limiting enzyme of the pentose phosphate pathway (PPP), shunting the carbon flow from glucose to ribose-5-phosphate, thus generating the reducing agent NADPH that is essential for maintaining cellular redox status. Citrate, the intermediate for fatty-acid synthesis, was also decreased in siMKP1-C1 and siMKP1-C2 cells (Figure 2B 2C). Furthermore, the reduced glutathione (GSH) level was also significantly reduced (Figure 2, 2B, 2C), indicating that MKP-1 also regulates the glutathione metabolism of tumor cells. Together, our data indicated that MKP-1 regulates metabolism essential for the proliferation and migration of NSCLC cells.

### MKP-1 positively regulates Nrf2 target genes

**Figure 3:**
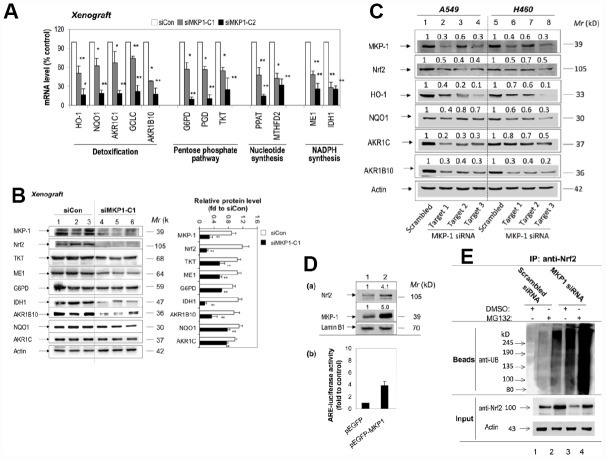
**MKP-1 regulates Nrf2 and its target genes in NSCLC xenograft tumors.** (**A**) and (**B**) Knockdown of MKP-1 reduces the expression of ARE-driven genes in NSCLC xenograft tumors. siMKP1-C1 or siCon cells were injected subcutaneously into Nu/Nu mice and the tumors were removed 6 weeks later. (**A**) mRNA levels of Nrf2 target genes determined by RT-PCR. The level of 18S rRNA was used as internal control. The value for siCon was set at 100%. Values are mean of three single tumours ± SD, n = 3 (**B**) Western immunoblots of the expression of MKP-1, Nrf2, and ARE-driven genes with antibodies against the indicated proteins. Each lane represents a single tumor. The relative levels of MKP-1, Nrf2, and ARE-driven genes normalized to actin are shown in right panel. The value for siCon was set at 1. Values are mean of three single tumours ± SD, n = 3. Blots are representative at least three separate experiments. (**C**) Knockdown of MKP-1 by siRNA against MKP-1 reduces the expression of Nrf2 protein and its target genes in A549 and H460 NSCLC cells. A549 and H460 cells were transfected with each of the MKP-1 siRNAs to Target 1, Target 2, or Target 3. Scrambled siRNA was transfected as negative control. Cells were harvested 48 h later and analyzed by Western immunoblotting with antibodies against the indicated proteins. The relative levels of MKP-1, Nrf2, and ARE-driven genes normalized to actin are shown above each lane. The value for scrambled siRNA-transfected cells was set at 1. Blots are representative at least three separate experiments. **p <0.01 (**D**) Overexpression MKP-1 increases the expression of Nrf2 protein and ARE-driven genes in A549 cells. (a) A549 cells were transfected with pEGFP-MKP1 or pEGFP vector 24 h before the cells were harvested. Nuclear extracts were probed by immunoblot with anti-MKP-1, anti-Nrf2, or anti-Lamin B1. The relative levels of MKP-1 or Nrf2 normalized to lamin B1 are shown above each lane. The value for pEGFP-transfected cells was set at 1. (b) Luciferase activity in A549 cells transfected with pEGFP, or pEGFP-MKP1. Co-transfections were performed with pGL-GSTA2.41bp-ARE reporter vector and pRL-TK. Dual luciferase activities were analysed. The value for cells transfected with pEGFP plus pGL-GSTA2.41bp-ARE reporter vector and pRL-TK was set at 1. Data are presented as the mean ± SD of triplicate experiments. (**E**) MKP-1 regulates Nrf2 ubiquitination. A549 cells were transfected with scrambled siRNA or MKP-1 siRNA for 48 h. The cells were exposed to MG132 (20 μM) for 4 h before whole-cell lysates were harvested and subjected to immunoprecipitation with Nrf2. After washing, the immunoprecipitates (Beads) were probed by immunoblotting with anti-UB. The input represents 10% of the total amount of cell lysate use for immunoprecipitation. Results are representative of three separate experiments. *p <0.05, **p < 0.01.

To investigate the effect of MKP-1 on Nc2 and ARE-driven gene expression, we analysed the expression profiles of siMKP1-C1, siMKP1-C2, and siCon cells by Western bloting and RT-PCR analysis. Strikingly, Nrf2 protein levels were reduced markedly in siMKP1-C1 and siMKP1-C2 cells (Figure 1B, lanes 2 and 3). Moreover, we found that the expression of metabolic enzymes, as well as detoxifying enzymes and antioxidant proteins (Figure 2C), which are the target genes of Nrf2, were widely downregulated in MKP-1-knockdown cells. Quantitative RT-PCR revealed a nearly identical reduction of the mRNA levels of G6PD, IDH1, PGD, PPAT, MTHFD2, TKT, ME1, AKR1B10, HO-1, NQO1, AKR1C1, and GCLC in siMKP1-C1 and siMKP1-C2 xenograft tumors (Figure 3A and Figure 2C). Accordingly, the protein levels of metabolic enzymes and cytoprotective proteins were decreased in the xenograft tumors (Figures 3B, 2C and Supplementary Figure 1). Furthermore, a prominent down-regulation of Nrf2 target genes at both protein levels and the mRNA was also observed in cultured siMKP1-C1 and siMKP1-C2 cells (Supplementary Figure 2A and 2B). These data uncovered a previously unidentified role of MKP-1, which functions as a key upstream regulator of multiple metabolic and cytoprotective genes in cancer, changes in whose expression in turn lead to metabolic outcomes beneficial to tumor growth.

### MKP-1 interacts with Nrf2 and promotes its stability

We next investigated how MKP-1 regulates Nrf2. RT-PCR analysis showed that MKP-1-knockdown failed to alter the Nrf2 mRNA levels in siMKP1-C1 and siMKP1-C2 cells or xenograft tumors obviously (data not shown). In contrast, Nrf2 protein diminished in siMKP1-C1 and siMKP1-C2 cells and xenografts (Figures 1B and 3B), suggesting that MKP-1 acts at the protein level. To further confirm the connection between MKP-1 and Nrf2, we used three independent MKP-1 siRNAs to knock down MKP-1 in both A549 and H460 NSCLC cells to minimize any off-target effects. In all cases, MKP-1-knockdown was concomitant with a reduction in Nrf2 abundance (Figure 3C, lanes 2-4 and 6-8). Expression of the Nrf2 target genes HO-1, NQO1, AKR1C, and AKR1B10 was also repressed at the same time (Figure 3C). Conversely, over-expression of MKP-1 by transient transfection of pEGFP-MKP1 into A549 cells resulted in elevation of the steady-state level of Nrf2 protein (Figure 3D, a, lane 2), associated with dramatic increases in ARE-luciferase activity (Figure 3D, b). It is conceivable that MKP-1 interferes with ubiquitination of Nrf2. Thus, endogenous Nrf2 ubiquitination in A549 and siRNA directed MKP-1 knockdown A549 cells was measured under basal conditions. Cells were treated with a proteasome inhibitor, MG132, for 4 hours to block ubiquitinated Nrf2 from degradation. The results show a reduced basal level of ubiquitin-conjugated Nrf2 in A549 cells, compared to that in MKP-1 knockdown cells (Figure 3E). Our results indicate that MKP-1 is able to prevent ubiquitination of Nrf2.

**Figure 4:**
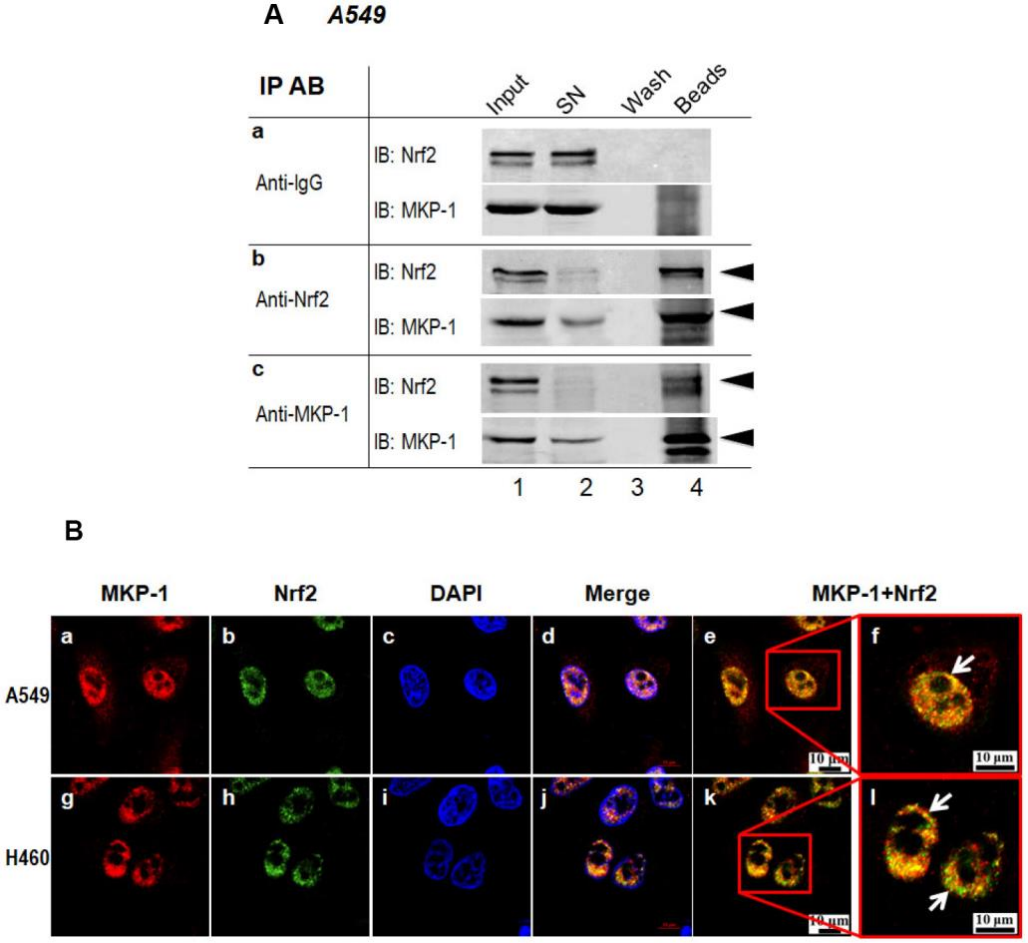
**MKP-1 interacts with Nrf2.** (**A**) Endogenous MKP-1 and Nrf2 interacted in A549 cells. Cell lysate from A549 cells was immunoprecipitated with antibody specific to MKP-1 or Nrf2. IgG was used as negative control. After washing, the immunoprecipitates were analysed by immunoblotting with antibody specific to Nrf2 or MKP-1. Input, 10% of the cell lysate used for immunoprecipitation. (**B**) Endogenous MKP-1 co-localised with Nrf2 in A549 and H460 cells. Cells were grown on cover-slips and fixed. Indirect immunofluorescence staining was performed to visualize endogenous MKP-1 using a primary rabbit antibody against MKP-1, and followed by Texas Red goat anti-rabbit secondary antibody. The endogenous Nrf2 was visualized using a primary mouse antibody against Nrf2 and followed by EGFP anti-mouse secondary antibody. The endogenous MKP-1 and Nrf2 are shown in red and green, respectively. Nuclei were stained with DAPI (blue). The scale bars represent 10 μm. Results are representative of three separate experiments.

In a very recent paper, we showed that MKP-1 associates with Nrf2 in response to phenolic antioxidant BHA, an Nrf2 activator [24]. To test whether MKP-1 is able to interact with Nrf2 directly, the whole-cell lysates from A549 cells were subjected to immunoprecipitation with anti-MKP-1 or anti-Nrf2 antibody. As seen, Nrf2 indeed formed complex with MKP-1 (Figure 4A, lane beads). Moreover, confocal laser scanning microscopy illustrated a similar nuclear co-localization of the two proteins in A549 and H460 cells (Figure 4B, image e, f, k and i). Thus, our data indicate that MKP-1 physically interacts with Nrf2 to prevent its uniquitination.

### MKP-1 is a target gene of Nrf2

**Figure 5:**
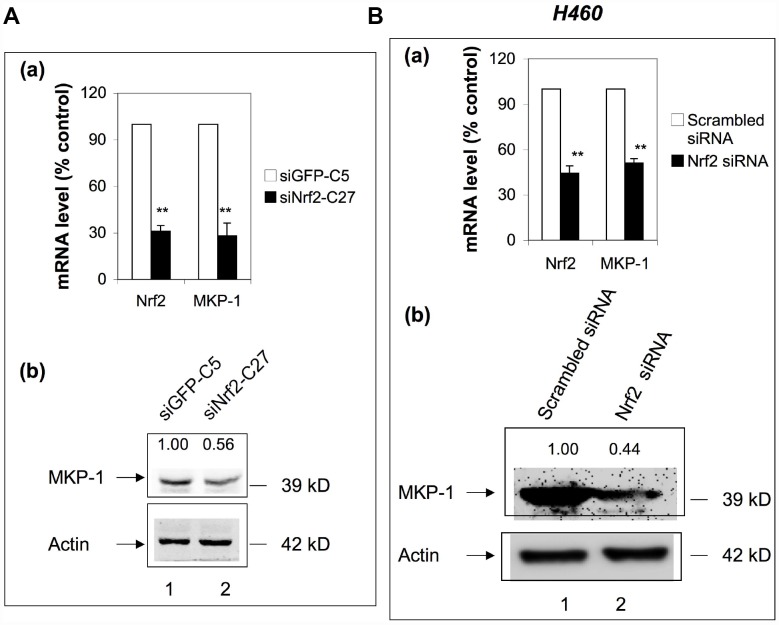
**MKP-1 is an Nrf2 target gene.** (**A**) Knockdown of Nrf2 reduced the expression of MKP-1 in A549 cells. siNrf2-C27 cells derived from A549 cells stably expressed siRNA against Nrf2. (a) mRNA levels of MKP-1 in siNrf2-C27 and siGFP-C5 cells were determined by RT-PCR. The level of 18S rRNA was used as internal control. The value for siCon cells was set at 100%. Data are presented as the mean ± SD of triplicate experiments. (b) MKP-1 protein expression in siNrf2-C27 and siGFP-C5 cells was determined by Western immunoblotting with anti-MKP-1. The relative levels of MKP-1 normalized to actin are shown above each lane. The value for siGFP-C5 cells was set at 1. (**B**) Knockdown of Nrf2 decreased the mRNA and protein levels of MKP-1 in H460 cells. H460 cells were transiently transfected with siRNA against Nrf2. (a) Total RNAs were harvested 24 h later. The mRNA levels of Nrf2 and MKP-1 were measured by RT-PCR. The level of 18S rRNA was used as internal control. The value for scrambled siRNA was set at 100%. (b) MKP-1 protein expression in cells transfected with scrambled siRNA or Nrf2-siRNA cells was determined by Western immunoblotting with anti-MKP-1. The relative levels of MKP-1 normalized to actin are shown above each lane. The value for scrambled siRNA-transfected cells was set at 1. Blots in 5Ab and 5Bb are representative at least three separate experiments. Data in 5Aa and 5Ba are presented as the mean ± SD of triplicate experiments (*p <0.05, **p <0.01).

To check whether Nrf2 has any feedback effect on the expression of MKP-1, we examined the expression of MKP-1 in siNrf2-C27 cells, an Nrf2 knockdown cell line derived from A549 [27]. Surprisingly, the MKP-1 mRNA level declined >70% compared to the control cell line, siGFP-C5 (Figure 5A, a), and the MKP-1 protein level was also reduced accordingly (Figure 5A, lane 2 of b). We also transiently transfected Nrf2-siRNA into NSCLC H460 cells to knock down Nrf2. Similarly, as a result, the MKP-1 mRNA and protein levels were reduced around 50% (Figure 5B), suggesting that MKP-1 is a target gene of Nrf2 in NSCLC cells.

### MKP-1 and Nrf2 expression are correlated in NSCLC biopsies

**Figure 6:**
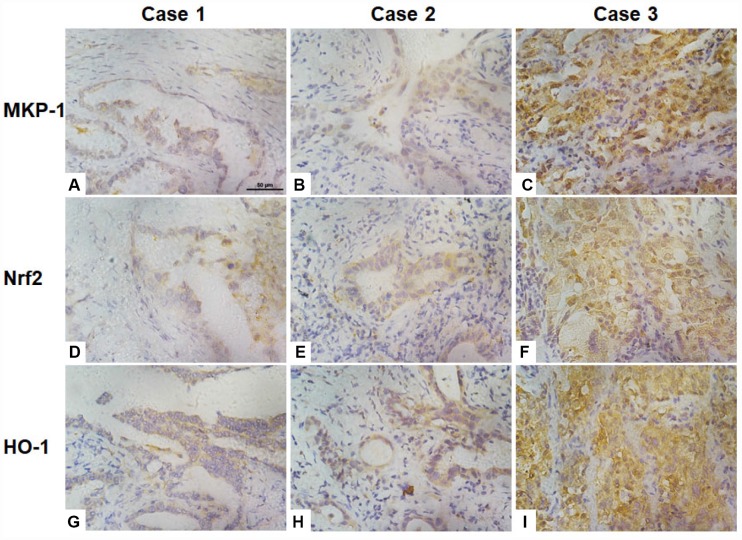
**Association of the expression of Nrf2, MKP-1, and HO-1 in human lung adenoma tissues.** Images of immunochemical staining for MKP-1 (**A**–**C**), Nrf2 (**D**–**F**), and HO-1 (**G**–**I**) from three representative lung adenoma tissues. Case 1, well-differentiated adenoma with weak positive staining for MKP-1 (**A**), Nrf2 (**D**), and HO-1 (**G**). Case 2, moderately-differentiated adenoma with moderate positive staining for MKP-1 (**B**), Nrf2 (**E**), and HO-1 (**H**). Case 3, poorly-differentiated adenoma with strong positive staining for MKP-1 (**C**), Nrf2 (**F**), and HO-1 (**I**). Original magnification ×400; scale bar, 50 μm.

To investigate correlations between the expression of MKP-1, Nrf2, and the Nrf2 target gene HO-1 in NSCLC cells, we assessed their immunohistochemical status in sections of tumor samples from 95 lung adenocarcinomas, of which >90% were moderately- or poorly-differentiated (Table 1 and Figure 6). MKP-1 and Nrf2 staining in tumor tissues were positive and mainly nuclear (Figure 6). In contrast, stain was localized both in the cytoplasm and nucleus in normal bronchial epithelium surrounding the tumor, showing weaker nuclear staining than in tumor tissue. HO-1 stain was localized in the cytoplasm in tumor tissue (Figure 6). High expression of MKP-1 was found in 50, Nrf2 in 57, and HO-1 in 57 of the 95 cases. As expected, the expression of Nrf2 was associated with HO-1 (r = 0.315, p <0.01). Interestingly, the expression of MKP-1 was associated with the expression of Nrf2 (r = 0.486, p <0.01) and with that of HO-1 (r = 0.227, p <0.05) (Table 2). In addition, we found that Nrf2 (p <0.05) and MKP-1 (p <0.01) were associated with patient age (≤60 years). The associations between immunohistochemical Nrf2, MKP-1, and HO-1 status and various clinicopathological parameters are summarized in Tables 1 and Supplementary Table 2. No significant association was detected between Nrf2 and MKP-1 and sex or tumor stage.

**Table 1:** Characteristics of 95 patients and samples in this study.

**Characteristics**	**No. of patients**	**Percentage (%)**
Age (years)		
Median (range)	61 (34–78)	
Sex		
Male	43	45.3
Female	52	55.9
Histological differentiation		
Poor	13	13.7
Poor-moderate	53	55.8
Moderate	22	23.2
Moderate-well	5	5.3
Well	2	2.1
MKP-1		
High^†^	50	52.6
Low^‡^	45	47.3
Nrf2		
High^†^	57	60
Low^‡^	38	40
HO-1		
High^†^	57	60
Low^‡^	38	40

^†^50% of cancer cells stained

^‡^<50% of cancer cells stained

**Table 2:** Correlation between expression of MKP-1, Nrf2, and HO-1 in the 95 human lung adenoma samples.

**Spearman correlation coefficient**	**MKP-1**	**Nrf2**	**HO-1**
MKP-1	1.000	0.486**	0.227*
Nrf2	0.486**	1.000	0.315**
HO-1	0.227*	0.315**	1.000

*p <0.05, **p <0.01 (two-tailed)

## DISCUSSION

In our recent work, we addressed the interplay between MKP-1 and Nrf2 in cytoprotective actions in mice liver and colon [24–26]. We have reported a new physiological role of MKP-1 in the maintenance of redox homeostasis in the liver. We also revealed a new mechanism by which MKP-1 upregulates Nrf2 through direct interaction with the transcription factor. Moreover, MKP-1 bound to the Neh2 domain of Nrf2 [24]. The present work expands such study to the NSCLC cells.

MAPKs play diverse and sometimes apparently contradictory roles in the initiation and development of lung cancers. In the case of the MKP-1 that negatively regulates MAPKs, there is increasing evidence that the enzyme may be abnormally regulated in lung cancer. However, the role of MKP-1 in tumourigenesis is as yet unclear [28]. Here, we uncovered novel functions and targets of MKP-1 in NSCLC cells. We showed in this study, that MKP-1 is a key player in regulating GSH biosynthesis as well as tumor metabolism that facilitates the biosynthesis of cellular building materials, providing proliferative advantages for cancer cells, sensitizing cells to anti-cancer drugs. Knockdown of MKP-1 resulted in decreased glucose consumption and lactate production, indicating reduced glycolysis and anabolism. Several enzymes involved in glucose uptake, glycolysis, PPP, lipid synthesis, glutamine metabolism, and the TCA cycle were prominently down-regulated in MKP-1 knockdown cells and xenograft tumors. In addition, glutathione biosynthesis was also down-regulated. Importantly, we demonstrated that MKP-1-mediated metabolic re-programming takes place through increased stability of Nrf2, which, in turn, controls the expression profiles of multiple key metabolic pathways in addition to cytoprotective genes.

MKP-1 gene is a transcriptional target of the p53 tumor suppressor [28] and is up-regulated in response to a variety of cellular stress conditions including oxidative stress, DNA-damaging agents, and hypoxia at levels found in solid tumors [29]. Here, we found that high levels of MKP-1 and Nrf2 with a statistically significant correlation were present in human NSCLC specimens. Thus, MKP-1 represents a new mechanism for the upregulation of Nrf2 in NSCLC. Furthermore, we showed that MKP-1 is an Nrf2 target gene, and the upregulation of Nrf2 further enhanced the expression of MKP-1. The upregulation of Nrf2 in tumors occurs through two distinct mechanisms: diminished Nrf2 turnover and augmented Nrf2 mRNA levels. The loss of inhibition by Keap1, caused by somatic mutations or epigenetic changes, results in increased Nrf2 stability in different types of human tumor [30]. Recently, Lien et al (2016) reported that PI(3)K/Akt stabilizes and activates Nrf2 via p21Cip1/WAF1 accumulation and GSK-3 inhibition in breast cancer cells [31]. DeNicola et al. (2011) reported that the oncogenes Kras, Braf, and Myc activate Nrf2 transcription during tumorigenesis [32]. Here, we have uncovered a novel pathway for Nrf2 stabilization in tumors. Therefore, we hypothesize that any dysregulation of MKP-1 or Nrf2 in tumor cells activates the MKP-1/Nrf2 loop to keep the Nrf2-ARE system constitutively active, regulating the expression of genes involved in the PPP, generation of NAPDH, and synthesis of purine nucleotides. This has also expanded our knowledge of MKP-1 functions from regulating MAPKs to the regulation of metabolism and the synthesis of macromolecules, thereby providing an explanation of how MKP-1 support cell proliferation and drug resistance.

Nrf2 is frequently deregulated in NSCLC through somatic mutations that disrupt the Nrf2-Keap1 interaction to constitutively activate Nrf2 [15, 33]. Recent report that investigated the Nrf2 and Keap1 protein levels in 304 NSCLC tissues found that 26% of the studied cohart had high nuclear Nrf2 levels, where 56% had low Keap1 levels [16]. Cancers with high Nrf2 levels are associated with poor prognosis [16, 17], resistance to clinical chemotherapy and rapid proliferation [17, 33]. Inhibiting the Nrf2-mediated protective mechanism to enhance the efficacy of cancer therapeutics represents a good approach to cancer treatment [18]. It is conceivable, given the ever expanding regulatory networks that surround Nrf2, that a wide range of potential Nrf2 inhibitor targets remain to be identified. Knowledge of these targets and the mechanism by which the regulate Nrf2 transcriptional activity would greatly accelerate efforts to discover the targets of novel Nrf2 inhibitors in the future [17, 27, 34–36].

## CONCLUSIONS

We have characterized in biochemical detail a cellular pathway in NSCLC cells, as initiated by MKP-1, which is important for the control of Nrf2, consequently the cytoprotective program and metabolism in cancer cells. Thus, in tumours in which Nrf2 is constitutively unregulated, inhibition of MKP-1 represents a potentially useful therapeutic approach to overcome drug resistance and inhibit cell proliferation in NSCLC.

## MATERIALS AND METHODS

### Chemicals

Unless otherwise stated, all chemicals were from Sigma-Aldrich Co., Ltd. Anti-Nrf2 (H300; sc-13032), Anti-Ub (sc-8017), anti-MKP-1 (sc-1102) were from Santa Cruz Biotechnology (Santa Cruz, CA, USA). Antiserum against aldoketo reductases 1C1 and C2 (AKR1C) was kindly provided by Professor John Hayes (University of Dundee, Dundee, UK). Anti-IDH1 (Cat#: 3997) were from Cell Signaling Technology (Danvers, MA, USA). Anti-TKT (Cat#: ab88438), anti-ME1 (Cat#: ab87974), and anti-G6PD (Cat#: ab993) were from Abcam (Cambridge, United Kingdom). Actin antibody (Cat#: R1207) and IgG antibody (Cat#: HA1011) were purchased from Hua-an (Hangzhou, Zhejiang, China). Antibodies against NQO1, HO-1 or AKR1B10 were raised in this laboratory and described previously [37, 38].

### Cell culture

NSCLC A549 and H460 cell lines were from the American Type Culture Collection. To generate stable MKP-1-knockdown lines, A549 cells were transfected with the pGFP-V-RS-MKP-1 plasmid expressing shRNA against human MKP-1. After selection in culture medium containing 0.5 μg/ml puromycin, two clones named siMKP1-C1 and siMKP1-C2, which maintained stable reduced expression of MKP-1 after multiple passages, were chosen for this study. Similarly, a cell line named siCon was generated after A549 cells were stably transfected with the empty pGFP-V-RS vector, and used as negative controls. The A549-derived Nrf2-knockdown cell line, siNrf2-C27, and its control cell line siGFP-C5, were described previously [27].

### *In vivo* tumor xenografts

Six-week-old BALB/c, nu/nu male nude mice were obtained from the Shanghai Laboratory Animal Center (Shanghai, China). Tumors were induced in the mice by inoculating ten million siCon, siMKP1-C1, or siMKP1-C2 cells per mouse (n = 8) as described previously [39]. Ten to fourteen days later, xenografts started growing. The sizes of tumors were measured twice weekly in two dimensions. Mice were sacrificed 6 weeks after inoculation. The tumors were excised and weights were recorded. The tissues were processed as described previously [36]. All animal procedures were performed with the approval of the Laboratory Animal Ethics Committee of Zhejiang University.

### siRNA, plasmids and transfections

Small interfering RNAs (siRNAs) and non-targeting negative control siRNA (scrambled-siRNA) were synthesized by TaKaRa Biotechnology (Dalian, China). The sequences for siRNAs against Nrf2 were as described previously [40]. The sequences for siRNAs against MKP-1 are provided in Supplementary Table 1.

pEGFP-MKP-1, expressing full-length human MKP-1 (RefSeq #: NM_004417.3), was made by PCR amplification from a human cDNA library (Clontech) and cloning in-frame into the *EcoR*I and *BamH*I sites of the pEGFP (Clontech) vector. Sequences of cloning primers are 5′-GAATTCCATGGTCATGGAAGTGG GCAC-3′ (forward) and 5′-GGATCCTCAGCAGCTG GGAGAGGTCGT-3′ (reverse). Plasmid pGFP-V-RS-MKP1 expressing shRNA against human MKP-1 was constructed. The sequence of MKP-1 shRNA was 5′-CCAAUUGUCCCAACCAUUUU-3′. All plasmids were verified by DNA sequencing. Lipofectamine 2000 (Invitrogen), was used for transfection of plasmids or siRNA. Empty vectors were used as negative controls for transfection experiments with plasmids. Twenty-four or 48 hours post-transfection, the transfected cells were harvested for further analysis.

### Real-time quantitative PCR (RT-qPCR)

Total RNA was prepared using TRIzol reagent (Invitrogen) and reverse transcribed using oligo-dT primer and SuperScript II reverse transcriptase (Invitrogen) as described previously [27]. qPCR using the validated SYBR® Green or TaqMan assays were carried out on a LightCycler® 480 instrument (Roche, Germany). All primers and probes were synthesized by TaKaRa Biotechnology. The primers and probes for detecting human Nrf2, HO-1, NQO1, AKR1C1, and GCLC were as described previously [27]. The sequences of the primers and probes for detecting human G6PD, IDH1, ME1, MTHFD2, PGD, PPAT, TKT, AKR1B10, and MKP-1 are listed in Supplementary Table 1.

### *In vivo* ubiquitination, immunoprecipitation and Western blot analysis

To detect endogenous Nrf2 that is ubiquitin-conjugated, cells were exposed to 10 μM MG132 (Sigma) for 4 hours prior to lysis. Cell lysates were subjected to immunoprecipitation with an anti-Nrf2 antibody and precipitated proteins were immunoblotted with an anti-Ub antibody. Immunoprecipitation procedures were performed as described previously [36]. Preparation of protein samples, SDS-PAGE gels, and immunoblotting were carried out as described previously [27, 41]. Immunoblotting with antibody against actin was performed to confirm equal loading of whole-cell extracts, while lamin B1 was used as loading control for nuclear extracts. The relative levels of the protein of interest were calculated by quantification of band intensity with an Odyssey infrared imaging system (LI-COR^®^ Biosciences) and normalized to actin or lamin B1.

### Cell proliferation, cytotoxicity, *in vitro* scratch migration, and Matrigel invasion assays

To monitor proliferation, cells were cultured in 6-well plates at 5 × 10^5^ cells per well. The cell proliferation were monitered after 24, 48, and 72 h. Toxicity and cell viability was monitored by MTS assays as described previously [36]. For *in vitro* scratch migration assays, cells were cultured in 24-well plates and scratched with the fine end of a 1-ml pipette tip (time 0). Plates were washed with PBS to remove detached cells and incubated with complete growth medium. Cell migration was photographed using 10 high-power fields, at time 0 and 24 h after injury. The rate of migration was measured by measuring the total distance that the cells moved from the edge of the scratch toward the center. Transwell analysis and quantification of migrated cells was performed as described previously [5]. All experiments were performed with 6–8 wells per experiment and repeated at least three times.

### Metabolic enzyme reactions and cellular GSH analysis

Cells were seeded at 1.2 × 10^5^ cells/well in a 12-well plate and cultured for three days before the culture media and cell pellets were harvested. The concentrations of glucose and lactate in the medium were determined using enzyme-based kits (BioVision, Inc.) (CA, USA) to estimate glucose consumption and lactate production. The G6PD activity and concentrations of citrate and NADPH in the cell pellets were determined using enzyme-based kits (BioVision, Inc.) following the manufacturer’s instructions. Cellular GSH analysis was as described elsewhere [42]. Determination of GSH Reduced glutathione was measured as described elsewhere [43]. Briefly, cells were seeded at 1 × 10^4^ cells per well in 96-well plates. After overnight incubation, cells were lysed. Cell lysates (100 μl) were incubated with 100 μl PBS containing 80 mM monochlorobimane (mCB) and 1 U/ml glutathione S-transferase for 1 hr at 25°C. Formation of the GS-mCB adduct was quantified by its fluorescence with excitation at 390 nm and emission at 490 nm.

### Patient samples and immunohistochemical (IHC) analysis

To determine the expression of MKP-1, Nrf2, and HO-1 in primary NSCLCs, we selected 95 archived, formalin-fixed, paraffin-embedded tumor tissue samples from surgically resected lung cancer specimens in the Cancer Tissue Bank at the First Affiliated Hospital, Zhejiang University School of Medicine, Hangzhou, China. The study was approved by the Ethics Committee of Zhejiang University School of Medicine. Tumor tissues were histologically analyzed and classified using the 2004 WHO classification system. All 95 tumors were histologically diagnosed as adenocarcinoma. The clinicopathological characteristics of the patients are summarized in Table 1. The male:female ratio was 43:52. None of these patients had received chemotherapy or radiotherapy before operation. The IHC analysis was carried out using standard procedures as described previously [37]. IHC expression was quantified by two independent pathologists, who were blinded to the clinical data. Nuclear Nrf2 and MKP-1, and cytoplasmic HO-1 expression was assessed based on both the proportion of positive cells and the intensity of staining. The extent of reactivity was evaluated and expressed as the percentage of positive cells, and classified into four grades: grade 0, <5%; grade 1, 5–25%; grade 2, 25–50%; grade 3, >50% positive cells. The extent of staining was quantified using a four-value scale: grade 0, none; grade 1, weak; grade 2, moderate; grade 3, strong staining. An immunohistochemical expression score was obtained by multiplying the intensity and reactivity values (range, 0–9), and these scores were used to determine the expression levels. The mean scores for Nrf2, MKP-1, and HO-1 expression in the tumor tissues from all lung cancer cases were 5, 5, and 4, respectively. For statistical analysis, high expression of nuclear Nrf2 or MKP-1 was defined as a score ≥5, while low expression was a score ≤ 4. High expression of cytoplasmic HO-1 was defined as a score ≥4, while low expression was a score ≤3.

### Statistical analysis

Statistical analysis was carried out using Stata7 for Windows (StataCorp LLC, College Station, TX, USA). Spearman’s correlation test was used to analyze two ranked variables. Statistical comparisons were performed using unpaired Student’s *t*-tests. A value of *p* <0.05 was considered statistically significant.

## Supplementary Material

Supplementary Figures

Supplementary Tables
