# Supplementary material for: Interplay of MKP-1 and Nrf2 drives tumor growth and drug resistance in non-small cell lung cancer
Source: Aging (Albany NY). 2019 Dec 6;11(23):11329–46. doi: 10.18632/aging.102531 (PMC6932920; doi:10.18632/aging.102531)
Supplement: Supplementary Figures [file aging-11-102531-s002..pdf]

SUPPLEMENTARY FIGURES

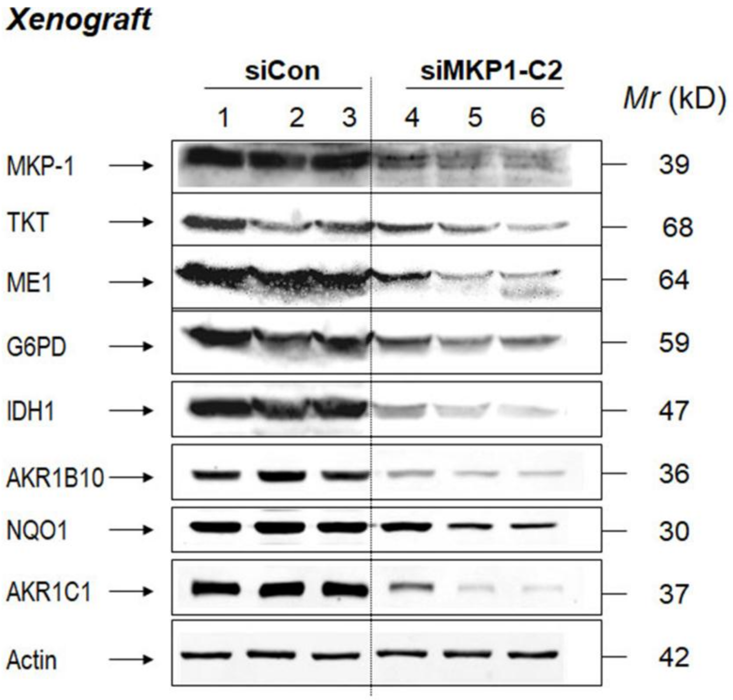

Supplementary Figure 1. MKP-1 regulates the expression of detoxifying and metabolic enzymes in NSCLC xenograft tumors. siMKP1-C2 or siCon cells were injected subcutaneously into Nu/Nu mice, and the tumors were removed 6 weeks later. The expression of MKP-1 and ARE-driven genes was determined by Western immunoblotting with antibodies specific to the indicated proteins. Each lane represents a single tumor.

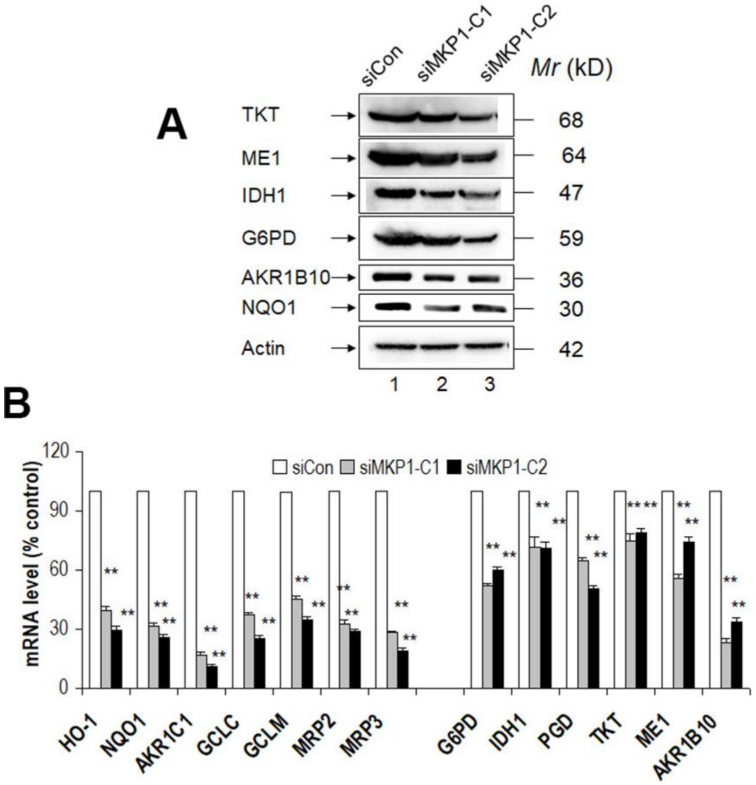

Supplementary Figure 2. MKP-1 regulates ARE-driven genes in cultured NSCLC cells. siMKP1-C1, siMKP1-C2, and siCon cells were seeded in 100-mm dishes in triplicate in DMEM plus 10% FBS. Forty-eight hours later the whole cell lysate was extracted from the subconfluent cells, while RNA was harvested from using Trizol (Invitrogen). (**A**) Expression of Nrf2 target genes TKT, ME1, IDH1, G6PD, AKR1B10, NQO1 in siMKP1-C1, siMKP1-C2, and siCon cells by Western immunoblotting with antibodies against the indicated proteins. Actin was used as a loading control. (**B**) mRNA levels of the ARE-driven genes analyzed by RT-PCR. The level of 18S rRNA was used as an internal control. The value for siCon was set at 100%. Data are presented as the mean ± SD of triplicate experiments. ***p* <0.01.
