# Supplementary material for: Interplay of MKP-1 and Nrf2 drives tumor growth and drug resistance in non-small cell lung cancer
Source: Aging (Albany NY). 2019 Dec 6;11(23):11329–46. doi: 10.18632/aging.102531 (PMC6932920; doi:10.18632/aging.102531)
Supplement: Supplementary Tables [file aging-11-102531-s001..pdf]

SUPPLEMENTARY TABLES

Supplementary Table 1. siRNA, PCR primers, and probes.

Primer name	Sequence (5’ → 3’)
siRNA	
MKP-1 (target 1)	F: CCAAUUGUCCCAACCAUUUU<br>R: AAAAUGGUUGGGACAAUUGG
MKP-1 (target 2)	F: GGAGGAUACGAAGCGUUUU<br>R: AAAACGCUUCGUAUCCUCC
MKP-1 (target 3)	F: GCAUCAUCUCUCCCAACUU<br>R: AAGUUGGGAGAGAUGAUGC
Primers for SYBR® Green RT-PCR assays	
G6PD	F: TGACCTGGCCAAGAAGAAGA<br>R: CAAAGAAGTCCTCCAGCTTG
IDH1	F: CACTACCGCATGTACCAGAAAGG<br>R: TCTGGTCCAGGCAAAAATGG
ME1	F: CTGCCTGTCATTCTGGATGT<br>R: ACCTCTTACTCTTCTCTGCC
MTHFD2	F: TGTCCTCAACAAAACCAGGG<br>R: TTCCTCTGAAATTGAAGCTGG
PGD	F: ATATAGGGACACCACAAGACGG<br>R: GCATGAGCGATGGGCCATA
PPAT	F: CGGTGCCAACATTCAAATC<br>R: TGGTGTGTCCAATTCCAAGA
TKT	F: GCTGAACCTGAGGAAGATCA<br>R: TGTCGAAGTATTTGCCGGTG
AKR1B10	F: 5′-TGTGGGCCTGGGCACTTGGA-3′<br>R: 5′-GGTCCTCCCGCTTCACAGCC-3′
Primers and probes for Taqman® RT-PCR analysis	
Nrf2	F: ACTCCCTGCAGCAAACAAGAG<br>R: TTTTTCTTAACATCTGGCTTCTTACTTTT<br>Probe: TGGCAATGTTTTCCTTGTTCC(5'-FAM 3'-BHQ1)
MKP-1	F: CACTGCCAGGCAGGCATT<br>R: CTCGATTAGTCCTCATAAGGTAAGCA<br>Probe: CCCGGTCAGCCACCATCTGCC(5'-FAM 3'-TAMRA)

Supplementary Table 2. Correlations of clinicopathological features with overexpression of MKP-1, Nrf2, and HO-1 in 95 patients with NSCLC.

Characteristics	Cases	MKP-1				Nrf2				HO-1			
		Low‡	High†	χ^2	*p* value	Low	High	χ^2	*p* value	Low	High	χ^2	*p* value
**Age (years)**													
≤60	43	13	30	9.253	**0.004****	11	32	6.805	**0.012***	15	28	0.857	0.404
≥61	52	32	20			27	25			23	29		
**Sex**													
Female	52	22	30	1.180	0.277	17	35	2.556	0.142	17	35	2.556	0.142
Male	43	23	20			21	22			21	22		
**Histological differentiation**													
Poor	13	6	7	0.762	0.943	6	7	4.159	0.474	7	6	7.493	0.113
Poor-moderate	53	27	26			20	33			20	33		
Moderate	22	9	13			9	13			9	13		
Moderate-well	5	2	3			1	4			0	5		
Well	2	1	1			2	0			2	0		

*p <0.05, **p <0.01 (two-tailed)
†50% of cancer cells stained
‡<50% of cancer cells stained
